# Applicability of Guideline-Based Aortic Intervention Thresholds in the Indian Population

**DOI:** 10.1055/a-2921-6578

**Published:** 2026-07-30

**Authors:** Nimrat Grewal, Mohammed Idhrees, Jedidiah Samraj, Bashi Velayudhan

**Affiliations:** 1 Department of Cardiothoracic SurgeryAmsterdam University Medical Center, University of AmsterdamAmsterdamNetherlands; 2 Aortic Institute 25047 Yale New Haven HospitalNew HavenConnecticutUnited States; 3 Institute of Cardiac and Aortic Disorders (ICAD)SRM Institutes for Medical Science (SIMS Hospital)ChennaiTamil NaduIndia

**Keywords:** aorta/aortic, body surface area, population anatomy, practice guidelines, aortic dissection

## Abstract

**Background:**

Clinical guidelines for thoracic aortopathy rely on absolute diameter thresholds to guide surveillance and prophylactic intervention. These thresholds, derived largely from Western cohorts, are applied globally, including in populations with smaller baseline vascular dimensions.

**Methods:**

We performed a counterfactual, denominator-based analysis comparing the distribution of ascending aortic diameter and its body surface area (BSA)-indexed equivalent in a South Asian general population ( *n* = 2,000) with diameters at presentation in a cohort of acute Stanford type A aortic dissection (ATAAD) patients ( *n* = 71), with Bayesian 95% credible intervals around exposure ratios.

**Results:**

The general population showed a narrow distribution (median 30.2 mm; 95th percentile 37.6 mm; mean indexed diameter 18.5 ± 3.1 mm/m ^2^ ), with under 1% reaching the 45 mm guideline threshold. Dissections occurred across a broad diameter range (median 52 mm; indexed median 28.0 mm/m ^2^ , interquartile range [IQR] 22.1–34.0 mm/m ^2^ ) and were disproportionately concentrated in the extreme right tail of both distributions.

**Conclusion:**

Guideline-based absolute diameter thresholds correspond to an extremely small fraction of this South Asian population, yet dissections remain concentrated in the extreme right tail, a pattern preserved and amplified after BSA indexing. These findings support population-aware interpretation of aortic size thresholds.

## Introduction

Clinical practice guidelines for thoracic aortic disease rely predominantly on absolute aortic diameter thresholds to guide prophylactic intervention. 1 These thresholds, derived largely from Western cohorts, have been widely adopted as global standards of care. Their apparent simplicity and reproducibility have contributed to their broad international implementation.

However, the generalizability of absolute diameter thresholds across populations with fundamentally different anthropometric and cardiovascular profiles remains uncertain. 2 Population-based imaging studies have consistently demonstrated that average ascending aortic diameters in South Asians are markedly smaller than those reported in Western cohorts. 3 4

The so-called “aortic size paradox,” first highlighted by analyses from the International Registry of Acute Aortic Dissection, demonstrated that a substantial proportion of acute dissections occur at diameters below guideline-recommended intervention thresholds. 5 Importantly, Paruchuri and colleagues elegantly showed that this apparent paradox is largely explained by the underlying population distribution of aortic size, in which the denominator of individuals with smaller aortas is overwhelmingly large. 6 Their work emphasized that the occurrence of dissection at smaller diameters does not, in itself, invalidate current surgical thresholds.

Nevertheless, these analyses were performed in predominantly Western populations. Whether the same conceptual framework applies when absolute thresholds are extrapolated to populations with systematically smaller baseline aortic dimensions has not been explored.

We, therefore, performed a counterfactual population-based analysis to assess how current guideline-recommended aortic intervention thresholds align with the distribution of ascending aortic dimensions in the Indian population. To provide clinical context, we additionally examined ascending aortic diameters at presentation in patients with acute Stanford type A aortic dissection (ATAAD).

## Methods

### Ethics

The study protocol was reviewed by the institutional ethics committee of ***. Informed consent was waived due to the retrospective nature of the study and the use of anonymized data. The study was conducted in accordance with the Declaration of Helsinki.

### General Population Cohort

The general population cohort comprised 2,000 consecutive adult individuals who underwent non-contrast thoracic or thoracoabdominal computed tomography (CT) imaging at SRM Institutes for Medical Science (SIMS) Hospital between January and December 2022 for non-cardiac clinical indications. Individuals younger than 18 years of age were excluded. Ascending aortic diameter was measured perpendicular to the long axis of the vessel at the point of maximal diameter on axial images, using an inner edge-to-inner edge convention, following the reference. 5 CT acquisition was non-contrast and non-ECG-gated, consistent with the standard imaging protocol for non-cardiac indications. Measurements were performed by trained investigators following a predefined protocol, with initial harmonization to ensure consistency.

### Dissection Cohort

A separate cohort of ATAAD patients was analyzed for contextual purposes. Ascending aortic diameter at presentation was extracted from preoperative contrast-enhanced, ECG-gated CT imaging at the same tertiary care hospital between 2010 and 2022, using the same axial, inner edge-to-inner edge measurement convention described above.

### Counterfactual Denominator-Based Analysis

Ascending aortic diameters in both cohorts were categorized into predefined diameter categories with increments of 5 mm, to reflect the full population distribution and align with guideline-relevant thresholds. For each category, the proportion of individuals in the general population (denominator) and the proportion of dissections (numerator) were calculated. 6 An exposure ratio was derived by dividing the percentage of dissections by the percentage of the general population in each category. This ratio reflects relative concentration of dissections across the diameter spectrum and does not represent absolute risk. Given the very small number of individuals with diameters ≥55 mm, categories ≥45 mm were used to represent the extreme right tail of the population distribution for primary interpretation.

## Results

### Population Distribution of Ascending Aortic Diameter

Baseline characteristics are shown in Table 1 . Dissection patients were younger than the general population cohort (mean age 47.5 ± 13.1 vs. 59.5 ± 16.6 years) and predominantly male (87.3% vs. 61.2%). Dissection patients were taller, heavier, and had a larger mean body surface area (BSA; 1.88 ± 0.24 vs. 1.66 ± 0.16 m ^2^ ). Hypertension was more prevalent in the dissection cohort (64.8% vs. 43.6%), while diabetes mellitus was less common (19.7% vs. 38.0%). Smoking was rare in the general population (0.5%) but present in 19.7% of dissection patients. Connective tissue disease was present in five dissection patients (7.0%) and bicuspid aortic valve in four (5.6%). The majority of dissections presented acutely (69.0%). Entry tear location and malperfusion territory were not systematically recorded in this retrospective registry.

**Table 1:** Baseline characteristics

Variable	General population ( *n* = 2,000)	ATAAD cohort ( *n* = 71 a )	*p* -Value
Age, mean ± SD (years)	59.5 ± 16.6	47.5 ± 13.1	<0.001
Male sex, *n* (%)	1,224 (61.2%)	62 (87.3%)	<0.001
Height, mean ± SD (cm)	160.2 ± 6.0	168.4 ± 10.2 b	<0.001
Weight, mean ± SD (kg)	62.3 ± 9.5	76.4 ± 16.9 b	<0.001
BSA, mean ± SD (m ^2^ )	1.66 ± 0.16	1.88 ± 0.24 b	<0.001
Hypertension, *n* (%)	873 (43.6%)	46 (64.8%)	0.001
Diabetes mellitus, *n* (%)	760 (38.0%)	14 (19.7%)	0.002
Smoking, *n* (%)	9 (0.5%)	14 (19.7%)	<0.001
Chronic kidney disease, *n* (%)	Not recorded	11 (15.5%)	–
Coronary artery disease, *n* (%)	Not recorded	12 (16.9%)	–
Connective tissue disease, *n* (%)	Not recorded	5 (7.0%)	–
Bicuspid aortic valve, *n* (%)	Not recorded	4 (5.6%)	–
Acute presentation, *n* (%)	N/A	49 (69.0%) c	–
Ascending aortic diameter, median (IQR)	30.2 (28.0–32.8) mm	52.0 (44.0–60.0) mm	<0.001
BSA-indexed diameter, median (IQR)	18.3 (16.6–20.1) mm/m ^2^	28.0 (22.1–34.0) mm/m ^2b^	<0.001
Entry tear location	N/A	Not available d	—
Malperfusion territory	N/A	Not available d	—

Abbreviations: ATAAD, acute Stanford type A aortic dissection; BSA, body surface area; IQR, interquartile range.

a *n* = 71 for aortic diameter; height/weight/BSA available for 61 patients.

b Based on 61 patients with available anthropometric data.

c Twenty-two patients (31.0%) had chronic presentation.

d Entry tear location and malperfusion territory were not systematically recorded in this retrospective registry.

Ascending aortic diameter in the general population ( *n* = 2,000) demonstrated a narrow distribution, with a median of 30.2 mm and an interquartile range (IQR) of 28.0 to 32.8 mm. The 90th, 95th, and 99th percentiles were 36.0 mm, 37.6 mm, and 41.2 mm, respectively. More than 99% of individuals had an ascending aortic diameter below 45 mm, and only two individuals (0.1%) had diameters of ≥55 mm ( Fig. 1 ).

**Fig. 1:**
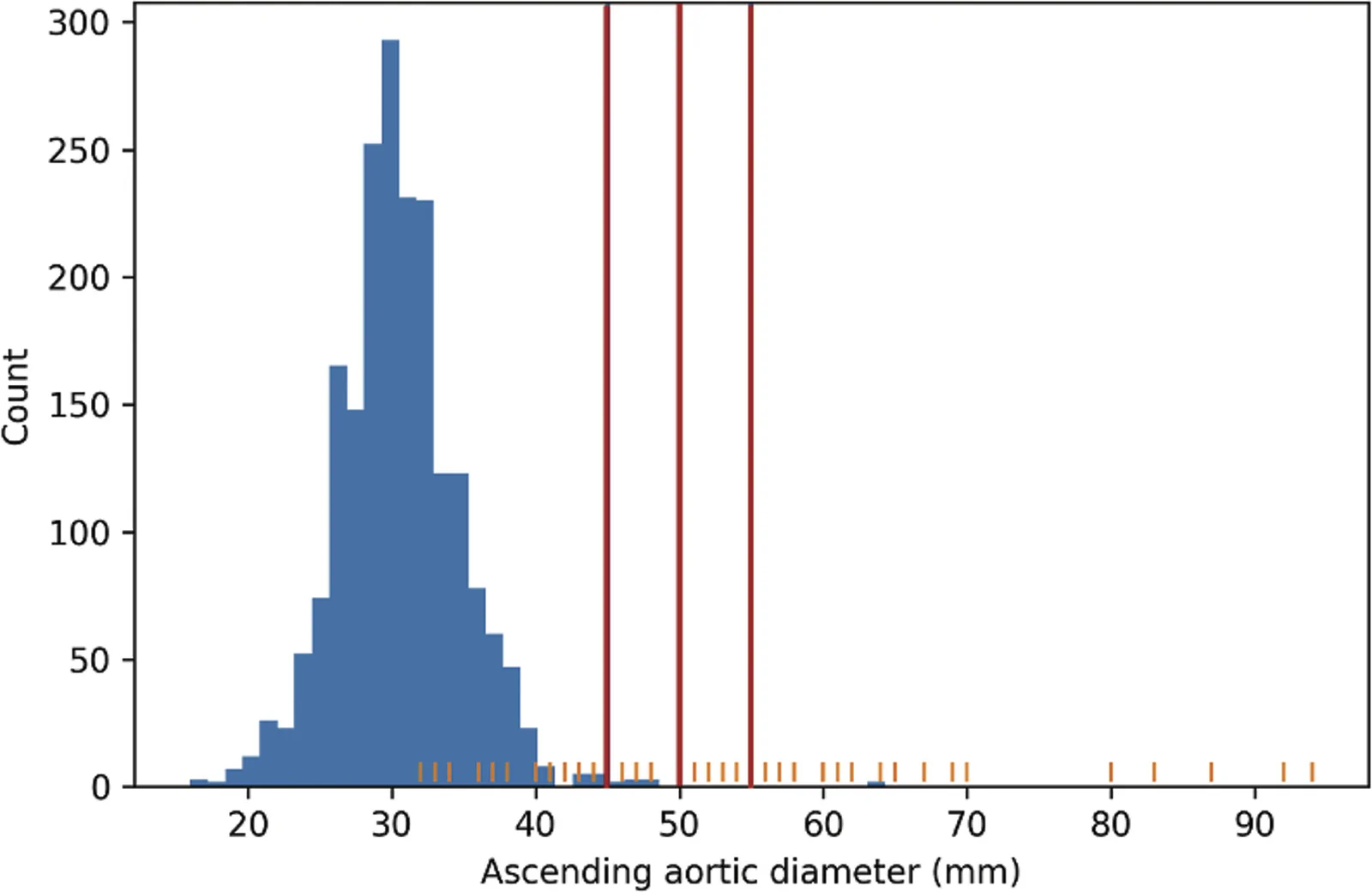
Distribution of ascending aortic diameters in the general population with guideline thresholds and dissections. Histogram showing the distribution of ascending aortic diameters in the general population. Vertical red lines indicate guideline-relevant thresholds (45, 50, and 55 mm). Individual ascending aortic diameters at presentation in acute Stanford type A aortic dissection are shown as rug marks along the x-axis. Multiple rug marks may overlap at identical diameter values. For visualization purposes, the histogram is truncated; dissection diameters beyond the displayed x-axis range are not shown.

### Ascending Aortic Diameter at Presentation in Acute Dissection

Ascending aortic diameter measurements were available for 71 ATAAD patients. The median diameter at presentation was 52 mm (IQR 44–60 mm), with values ranging from 32 to 94 mm. Nineteen dissections (26.5%) occurred at diameters below 55 mm, and nine (12.9%) occurred below 45 mm.

BSA was available for all 2,000 general population participants and was calculable (Mosteller formula) for 61 of the 71 dissection patients with ascending aortic diameter measurements. In the general population, the mean BSA-indexed ascending aortic diameter was 18.5 ± 3.1 mm/m ^2^ (median 18.3 mm/m ^2^ ; 95th percentile 23.4 mm/m ^2^ ; 99th percentile 27.0 mm/m ^2^ ). In the dissection cohort, the median indexed diameter was 28.0 mm/m ^2^ (IQR 22.1–34.0 mm/m ^2^ ; range 14.5–55.7 mm/m ^2^ ). The denominator-based concentration of dissections in the extreme right tail of the indexed distribution mirrored the pattern observed for absolute diameter ( Table 2 ): Less than 0.3% of the general population had an indexed diameter ≥30 mm/m ^2^ , yet this category accounted for 36.1% of dissections (exposure ratio 120.2; 95% Bayesian credible interval 53.2–301.8).

**Table 2:** Distribution of body surface area-indexed ascending aortic diameter in the general population and the acute Stanford type A aortic dissection cohort, with corresponding exposure ratios and Bayesian 95% credible intervals

Category (mm/m ^2^ )	Population, *n* (%)	Dissections, *n* (%)	Exposure ratio	95% Bayesian CrI
<15	205 (10.3)	1 (1.6)	0.2	0.02–0.73
15–19	1,274 (63.7)	6 (9.8)	0.2	0.07–0.30
20–24	479 (24.0)	18 (29.5)	1.2	0.80–1.76
25–29	36 (1.8)	14 (23.0)	12.8	7.04–21.75
≥30	6 (0.3)	22 (36.1)	120.2	53.24–301.77

Abbreviations: BSA, body surface area; CrI, credible interval.

Note: BSA-indexed ascending aortic diameter categories (mm/m ^2^ ) are shown for the general population (denominator; *n* = 2,000) and for patients with acute Stanford type A aortic dissection (numerator; *n* = 61 with available height and weight). BSA was calculated using the Mosteller formula. Percentages are calculated within each cohort. The exposure ratio represents the percentage of dissections divided by the percentage of the general population within each category and reflects relative concentration rather than absolute risk. Bayesian 95% credible intervals were derived using a Jeffreys-prior β-binomial model (1,000,000 Monte Carlo draws).

### Modeled Predissection Diameter

Ascending aortic diameter at presentation reflects the postdissection state. Applying the literature-derived correction factor of approximately 30% reported by Rylski et al (dividing postdissection diameters by 1.30), 7 the modeled median predissection diameter in our cohort was 40.0 mm (modeled IQR 33.8–46.2 mm; modeled range 24.6–72.3 mm). This modeled median exceeds the 95th percentile of the general population (37.6 mm), indicating that the concentration of dissections in the right tail of the population distribution is not solely attributable to postdissection diameter expansion.

### Sensitivity and Specificity of Guideline Thresholds

Treating the dissection cohort as cases and the general population as non-cases, sensitivity and specificity were calculated for four absolute diameter thresholds and three BSA-indexed thresholds ( Table 3 ). At the 45-mm threshold, sensitivity was 71.8% and specificity 99.5%; at 55 mm, sensitivity fell to 42.3% with specificity 99.9%. For indexed diameter, a 24 mm/m ^2^ threshold achieved sensitivity of 67.2% and specificity of 96.7%. Positive and negative predictive values were not estimated because population-level ATAAD incidence in this South Asian population has not been established.

**Table 3:** Sensitivity and specificity of absolute and body surface area-indexed ascending aortic diameter thresholds for identifying acute Stanford type A aortic dissection

Threshold	Population ≥ threshold	Dissections ≥ threshold	Sensitivity	Specificity
≥40 mm	29/2,000 (1.5%)	63/71 (88.7%)	88.7%	98.5%
≥45 mm	10/2,000 (0.5%)	51/71 (71.8%)	71.8%	99.5%
≥50 mm	2/2,000 (0.1%)	42/71 (59.2%)	59.2%	99.9%
≥55 mm	2/2,000 (0.1%)	30/71 (42.3%)	42.3%	99.9%
≥24 mm/m ^2^	66/2,000 (3.3%)	41/61 (67.2%)	67.2%	96.7%
≥28 mm/m ^2^	13/2,000 (0.7%)	31/61 (50.8%)	50.8%	99.3%
≥30 mm/m ^2^	6/2,000 (0.3%)	22/61 (36.1%)	36.1%	99.7%

Note: Cases, acute Stanford type A aortic dissection cohort ( *n* = 71 for absolute diameter thresholds; *n* = 61 with available body surface area for indexed diameter thresholds). Non-cases, general population cohort ( *n* = 2,000). Sensitivity represents the proportion of dissection patients at or above each threshold; specificity represents the proportion of the general population below each threshold. Positive and negative predictive values were not calculated, as the population-level incidence of acute type A aortic dissection in this South Asian population has not been established. Body surface area-indexed thresholds are expressed in mm/m ^2^ .

### Denominator-Based Distribution across Diameter Categories

Marked divergence between population anatomy and dissection distribution was observed across diameter categories ( Table 4 and Fig. 2 ). Categories below 40 mm accounted for over 98% of the general population but only 11.3% of dissections, yielding exposure ratios well below 1.

**Fig. 2:**
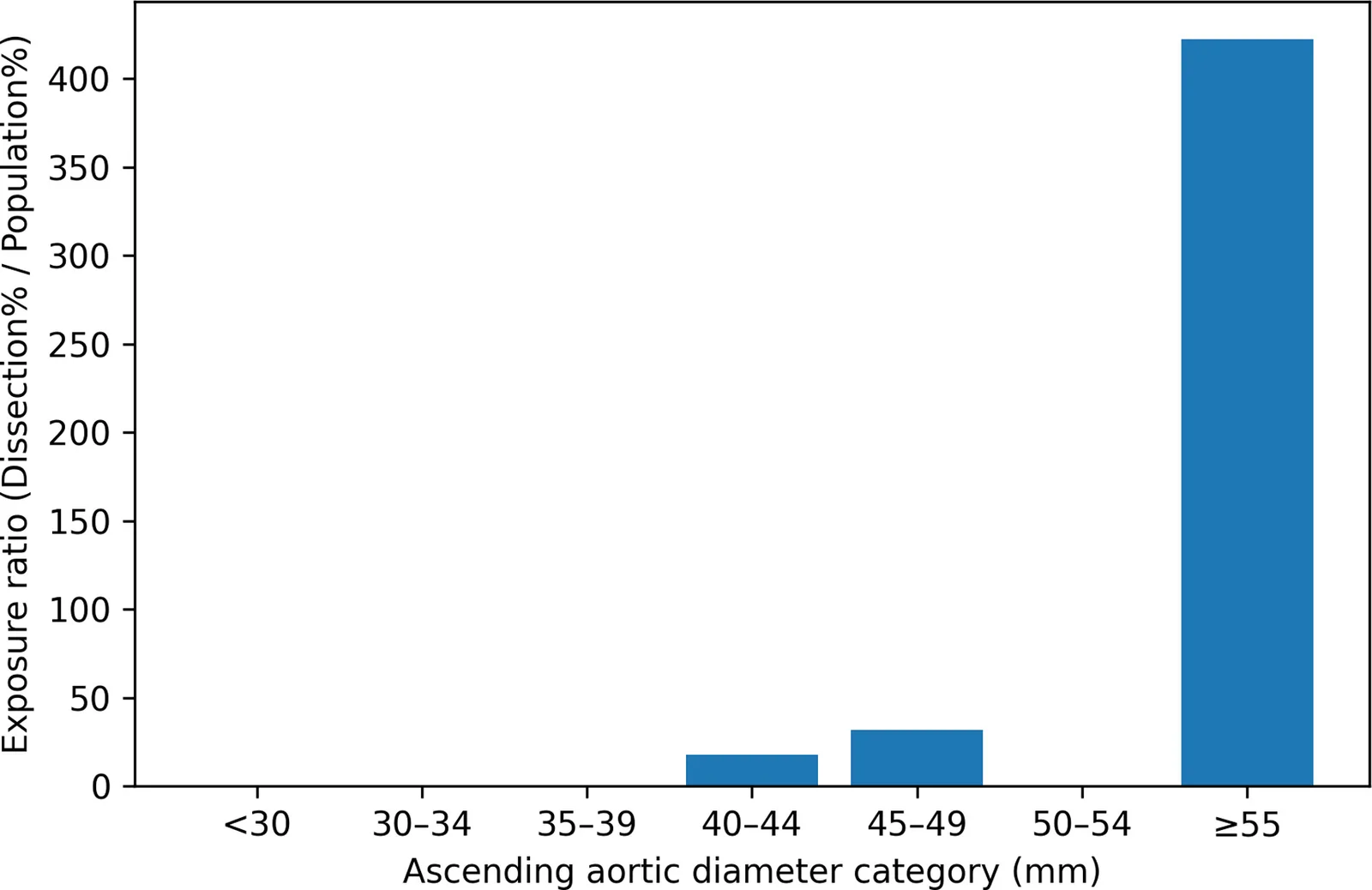
Denominator-based exposure of acute aortic dissection across diameter categories. Bar plot showing the exposure ratio for acute Stanford type A aortic dissection across ascending aortic diameter categories. The exposure ratio was calculated as the percentage of dissections divided by the percentage of the general population within each category and reflects relative concentration rather than absolute risk. Exposure ratios were not estimable for categories with a zero population denominator (50–54 mm).

**Table 4:** Distribution of ascending aortic diameter in the general population and the acute Stanford type A aortic dissection cohort, with corresponding exposure ratios and Bayesian 95% credible intervals

Category (mm)	Population, *n* (%)	Dissections, *n* (%)	Exposure ratio	95% Bayesian CrI
<30	914 (45.7)	0 (0)	0.0	0.00–0.08
30–34	829 (41.5)	4 (5.6)	0.14	0.05–0.31
35–39	228 (11.4)	4 (5.6)	0.49	0.17–1.14
40–44	19 (1.0)	12 (16.9)	17.8	8.77–34.54
45–49	8 (0.4)	9 (12.7)	31.7	12.57–79.98
50–54	0 (0)	12 (16.9)	Not estimable	Very wide; not reliably estimable (denominator = 0)
≥55	2 (0.1)	30 (42.3)	422.5	126.04–2,060.05

Abbreviation: CrI, credible interval.

Note: Ascending aortic diameter categories (mm) for the general population (denominator; *n* = 2,000) and acute Stanford type A aortic dissection patients (numerator; *n* = 71). Percentages are calculated within each cohort. The exposure ratio reflects relative concentration rather than absolute risk. Bayesian 95% credible intervals were derived using a Jeffreys-prior β-binomial model (1,000,000 Monte Carlo draws). The 50–54 mm exposure ratio was not estimable due to the zero population denominator.

In contrast, the 40–44 mm category represented 0.95% of the population but accounted for 16.9% of dissections (exposure ratio 17.8). Further enrichment was observed in the 45–49 mm category, which comprised 0.40% of the population and 12.7% of dissections (exposure ratio 31.7). Although extreme enrichment was observed in the ≥55 mm category, interpretation was limited by the very small number of individuals in the general population within this range. To quantify uncertainty around these estimates, Bayesian 95% credible intervals were calculated for each diameter category ( Table 4 ); intervals were appropriately wide in sparse right-tail categories, particularly for the 50–54 mm absolute category where the population denominator was zero.

These findings indicate that dissections are disproportionately concentrated in the extreme right tail of the population distribution, despite occurring across a broad absolute range of diameters.

## Discussion

In this counterfactual population-based analysis, we demonstrate a pronounced mismatch between guideline-based absolute aortic intervention thresholds and the distribution of ascending aortic dimensions in the Indian general population. In this cohort, commonly applied thresholds lie far beyond the anatomical range encountered in the vast majority of individuals, a population-anatomy observation that should not, on its own, be interpreted as evidence that the thresholds themselves are clinically inappropriate.

At the same time, denominator-based analysis provides essential context for interpreting the aortic size paradox. Although a substantial proportion of dissections occurred at diameters below guideline thresholds, dissections were overwhelmingly concentrated in the extreme right tail of the population distribution, where the denominator of exposed individuals is exceedingly small. This explains why the occurrence of dissections at smaller diameters does not, in itself, invalidate size-based intervention thresholds at a population level.

However, our findings also highlight an important limitation of absolute diameter as a universal surrogate for biological risk. In populations with smaller baseline anatomy, absolute thresholds identify only a vanishingly small fraction of individuals, while clinically relevant events occur across a broader relative range of aortic sizes. Under such circumstances, the discriminative value of absolute diameter alone diminishes.

These data do not argue for lowering surgical thresholds. Rather, they emphasize the need for population-aware interpretation of absolute aortic size. Relative approaches, such as indexed diameters, percentile-based assessment, or evaluation of deviation from expected size across the life course, may offer more informative frameworks for risk stratification in populations with smaller baseline aortic dimensions.

The BSA-indexed diameter analysis reinforces these findings. Despite having a larger absolute BSA than the general population cohort, dissection patients had a median indexed diameter (28.0 mm/m ^2^ ) that exceeded the 99th percentile of the general population (27.0 mm/m ^2^ ). An indexed threshold of 24 mm/m ^2^ , corresponding approximately to the 95th percentile in this South Asian population, achieved sensitivity of 67.2% and specificity of 96.7%, suggesting that population-specific indexed thresholds merit further prospective investigation.

As cardiovascular guidelines continue to be applied globally, explicit consideration of population anatomy and denominator effects is essential to ensure biologically meaningful and equitable implementation. Future work integrating cohort-specific operative outcomes within a decision-analytic framework, and prospective validation of population-specific or indexed thresholds, will be necessary to translate these descriptive findings into actionable clinical guidance.

## Limitations

Several limitations should be considered. First, the general population cohort comprised individuals undergoing CT imaging for non-cardiac indications at a single tertiary referral center, rather than a randomly sampled community-based cohort; this may introduce selection bias related to comorbidity or referral patterns. Second, the dissection cohort was relatively small ( *n* = 71), and exposure ratios in the extreme right tail should be interpreted descriptively even with Bayesian credible interval quantification. Third, ascending aortic diameter in the dissection cohort reflects the post-dissection state; based on the average diameter increase of approximately 30% at the time of dissection, modeled pre-dissection diameters in our cohort were likely smaller (modeled median ∼40.0 mm), though still at or above the 95th percentile of the general population. Predissection imaging was unavailable for all 71 patients. Fourth, ascending aortic length was not systematically recorded. The BSA-indexed analysis ( Table 2 ) addresses part of this limitation and confirms that the denominator-based concentration of dissections in the right tail is preserved after body-size adjustment.
